# A Hybrid Solid‐State NMR and Electron Microscopy Structure‐Determination Protocol for Engineering Advanced *para*‐Crystalline Optical Materials

**DOI:** 10.1002/chem.201700324

**Published:** 2017-02-16

**Authors:** Brijith Thomas, Jeroen Rombouts, Gert T. Oostergetel, Karthick B. S. S. Gupta, Francesco Buda, Koop Lammertsma, Romano Orru, Huub J. M. de Groot

**Affiliations:** ^1^ Leiden Institute of ChemistryEinsteinweg 552333CCLeidenThe Netherlands; ^2^ Vrije University AmsterdamDe Boelelaan 10831081 HVAmsterdamThe Netherlands; ^3^ Groningen Biomolecular Sciences and Biotechnology InstituteNijenborgh 79747 AGGroningenThe Netherlands; ^4^ Department of ChemistryUniversity of JohannesburgAuckland ParkJohannesburg2006South Africa

**Keywords:** electron microscopy, magic-angle spinning, NMR spectroscopy, photochemistry, self-assembly

## Abstract

Hybrid magic‐angle spinning (MAS) NMR spectroscopy and TEM were demonstrated for de novo structure determination of *para*‐crystalline materials with a bioinspired fused naphthalene diimide (NDI)–salphen–phenazine prototype light‐harvesting compound. Starting from chiral building blocks with *C*_2_ molecular symmetry, the asymmetric unit was determined by MAS NMR spectroscopy, index low‐resolution TEM diffraction data, and resolve reflection conditions, and for the first time the ability to determine the space group from reciprocal space data using this hybrid approach was shown. Transfer of molecular *C*_2_ symmetry into *P*2/*c* packing symmetry provided a connection across length scales to overcome both lack of long‐range order and missing diffraction‐phase information. Refinement with heteronuclear distance constraints confirmed the racemic *P*2/*c* packing that was scaffolded by molecular recognition of salphen zinc in a pseudo‐octahedral environment with bromide and with alkyl chains folding along the phenazine. The NDI light‐harvesting stacks ran orthogonal to the intermolecular electric dipole moment present in the solid. Finally, the orientation of flexible lamellae on an electrode surface was determined.

Chemical self‐assembly to bridge the gap from dead to living matter is a challenging field. Controlling complexity, flexibility, and functionality of synthetic and biomimetic material requires^1^ engineering soft *para*‐crystalline compounds that lack crystalline long‐range order in at least one dimension. However, resolving their microscopic and mesoscopic order at atomic resolution from the anisotropic background heterogeneity has proven to be quite difficult. Only recently has the hybrid bottom‐up magic‐angle spinning (MAS) NMR spectroscopy and top‐down electron microscopy (EM) structure‐determination methodology shown promise to overcome the limitations of either technique.^2^ MAS NMR spectroscopy is intrinsically a microscopic method^3^ that cannot solve a structure de novo because packing order is determined by minimizing steric hindrance with screw axes or glide planes at higher levels in the structural hierarchy. For small organic molecules forming microcrystals, modeling protocols with a conjectured rather than determined space group are used, making MAS NMR spectroscopy a chemical shift filter for selection and validation.

Earlier, we showed for the largest biological (protein‐free and grossly heterogeneous) light antennae that the limitations of MAS NMR spectroscopy can be diminished by using a 2D TEM periodogram as a band‐pass filter to resolve sparse regions of nonzero intensity in reciprocal space.^4^ Here we present the important next step by showing that genuine space‐group information can be extracted from TEM data in reciprocal space.

To demonstrate this, we use a prototypical engineered biomimetic chromophore light‐harvesting material that consists of fused Br‐substituted naphthalene diimide (NDI), phenazine, and Zn‐salphen building blocks (Figure 1).^5^ This system, denoted as DATZnS(3′‐NMe), models the chlorosome that is built from parallel stacks of bacteriochlorophyll (BChl) connected by metal coordination in a recognition motif. The BChl stacks form polarizable curved sheets and tubes that are thought to yield a dielectric response upon excitation with crossing of energy levels and coherent mixing of exciton states for energy transport. Although the model is chemically unrelated to BChl, we show that it self‐assembles into stacks that form extended polar curved sheets like the natural paradigm. The NDI of the model is capable of overlap, the phenazine carries the electric dipole, and the Zn‐salphen provides a recognition motif for coordination.^6^

**Figure 1:**
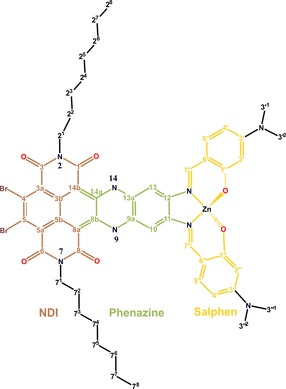
Chemical structure of *anti* DATZnS(3′‐NMe), a fused hybrid of NDI (red), phenazine (green), and salphen (yellow).

The molecular symmetry and asymmetric unit were determined from MAS NMR shifts collected with ^13^C at natural abundance. We indexed the TEM diffraction and determined both the unit‐cell parameters and a genuine space group by analysis of systematic absences in a sparse reciprocal space‐intensity pattern with the help of the molecular‐symmetry information obtained by MAS NMR spectroscopy. The technologies were then merged at the molecular level and unit cell. Because indexing of an unknown structure is not possible by TEM alone, the diffraction‐phase problem was implicitly overcome by connecting across length scales through transfer of molecular symmetry to packing symmetry. This provided a structural underpinning for engineering supramolecular material in the desired sheets with parallel aligned dipoles. Fourier‐transform filtering in reciprocal space averaged static heterogeneity in real space and enabled extrapolating the short‐ and medium‐range ordering in a *para*‐crystalline lattice to establish a full 3D lattice model, which we refined with MAS NMR heteronuclear ^1^H‐^13^C correlation data. Finally, we determined the orientation of DATZnS(3′‐NMe) on a surface to relate to the geometry of biomimetic material in electrode applications.

The symmetric NMR response of the DATZnS(3′‐NMe) provided conclusive evidence that the asymmetric unit was half of the molecule (Figure S1, Table S1 in the Supporting Information). Of the two possible configurations, the *syn* form with a mirror plane running along the center of the phenazine motif was approximately 10 kcal mol^−1^ less stable than the *anti* form that had a twofold axis.^7^ This was the first step in our approach.

High‐resolution TEM of the system on a carbon grid revealed curved lamellae (Figure 2 A). The Fourier transform showed strong centrosymmetric reflections at 1/1.685 nm^−1^ and perpendicularly a series of spots at 1/0.547 nm^−1^ with a systematic absence indicating *h*0*l* (*l*=2*n*) reflections (Figure 2 B). Both strong features were attributed to first‐order reflections and pointed to the molecule along the 1.685 nm direction with its *C*2 axis representing *P*2 packing symmetry. The systematic absence revealed an additional translation with a screw axis or glide plane. A screw in a different direction would imply an orthorhombic cell, in contrast with EM images that revealed a monoclinic cell with intensity at 1/1.24 nm^−1^ that was attributed to second‐order reflections for a realistic density of 1.67 g cm^−2^ (Figure S5 in the Supporting Information). This left a glide plane that explained both the systematic absence at 1/0.547 nm^−1^ in Figure 2 and the absence of first‐order reflections in Figure S5 in the Supporting Information. The mirror operation produced a racemic packing, with the wings of the salphen forming enantiomeric chiral Λ and Δ pairs.^10^ This led to a *P*2/*c* space group with four inequivalent sites, two from the twofold axis in the DATZnS(3′‐NMe) and two from the enantiomeric pair. This represents the second step of our approach, in which we overcame the diffraction‐phase problem and indexed the TEM data to resolve a genuine space group. Considering the weakness of the reflection spots in Figure S5 in the Supporting Information, there could be other polymorphs as well, but these did not pass the TEM diffraction filter.

**Figure 2:**
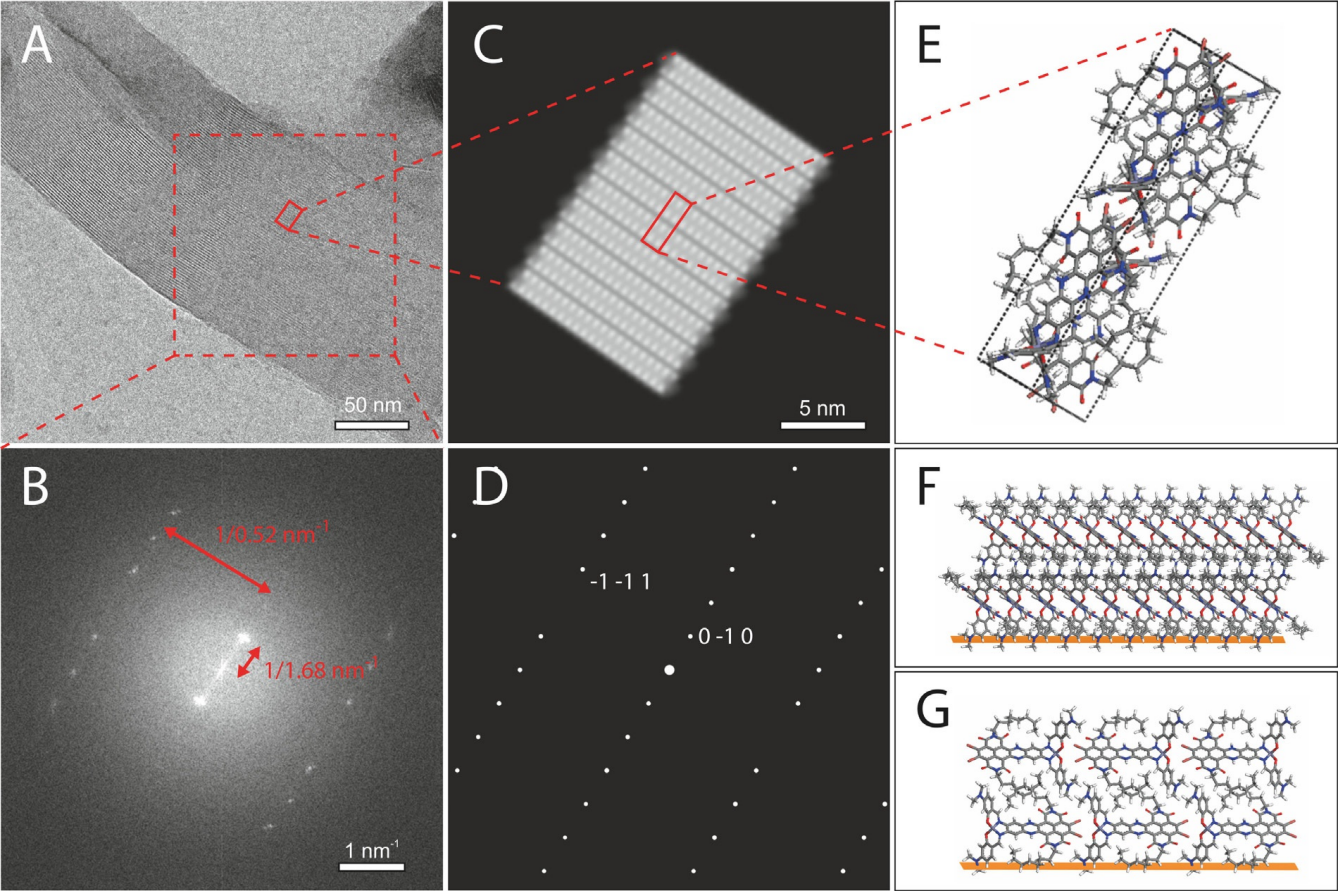
Integration of TEM and MAS NMR. (A) TEM image of the DATZnS(3′‐NMe) on a carbon‐coated grid, revealing the curved lamellar character of the compound; (B) Fourier transform of the selected region showing the TEM diffraction pattern with systematic absences; (C) projection of the electron‐density map with a 20×10×5 supercell;^8^ (D) simulated diffraction pattern obtained with the NMR‐derived geometry in the *P*2/*c* space group;^9^ (E) orientation of DATZnS(3′‐NMe) on the carbon grid (orange color); (F) viewed along the *b* axis; and (G) along the *a* axis.

The *P*2/*c* structure was modeled with unit‐cell dimensions *a=*0.547 nm, *b=*1.685 nm, *c=*2.517 nm, and *β=*102°, determined with TEM to an energy of 170.5 kcal mol^−1^ (see S10 in the Supporting Information for details). To validate the structure, we performed an optimization without constraining the cell, which led to virtually the same result. Next, long‐range transfer signals were identified in a heteronuclear ^1^H‐^13^C dataset recorded with a long contact time of 4 ms by comparing with data collected for a short mixing time (Figure S3 in the Supporting Information) and observed between the 3′^1^, 3′^2^, 3′′^1^, and 3′′^2^ protons of salphen and the 4, 5 ^13^C nuclei in the NDI motif (Figure 3). Transfer involving 3′‐NMe and 4, 5 ^13^C provided strong NMR evidence for molecular recognition between the NDI part of the molecule and the salphen motif of an adjacent molecule. The transfer of polarization, that is observed between protons on the alkyl chain and the 11, 12, 3*b*, 5*b*, 13*a*, 9*a*, 14*b*, 8*a*, 10, and 13 ^13^C nuclei on the phenazine backbone, positions the alkyl chain in the packing. The buildup of Lee‐Goldburg cross‐polaization (LGCP) signals for the 4, 5 and 13*a*, and 9*a* ^13^C nuclei from Figure S4 in the Supporting Information was in line with a simulation of transfer over approximately 4 Å. (Figure 4).^11^ The 3*b*, 5*b* ^13^C nuclei in the central part of the NDI motif correlated with the 2^1^ and 7^1^ CH_2_ protons. This revealed the formation of slipped *J*‐aggregates for the NDI, with the alkyl tail above the plane of a neighbouring molecule. It implied that the correlations and buildup from protons at the alkyl chain to 13*a*, 9*a* ^13^C nuclei were also intermolecular. Similarly, buildup of CP intensity from the alkyl CH_2_ to the quaternary ^13^C on the phenazine core could be considered intermolecular from the abundant cloud of protons on the alkyl chain.

**Figure 3:**
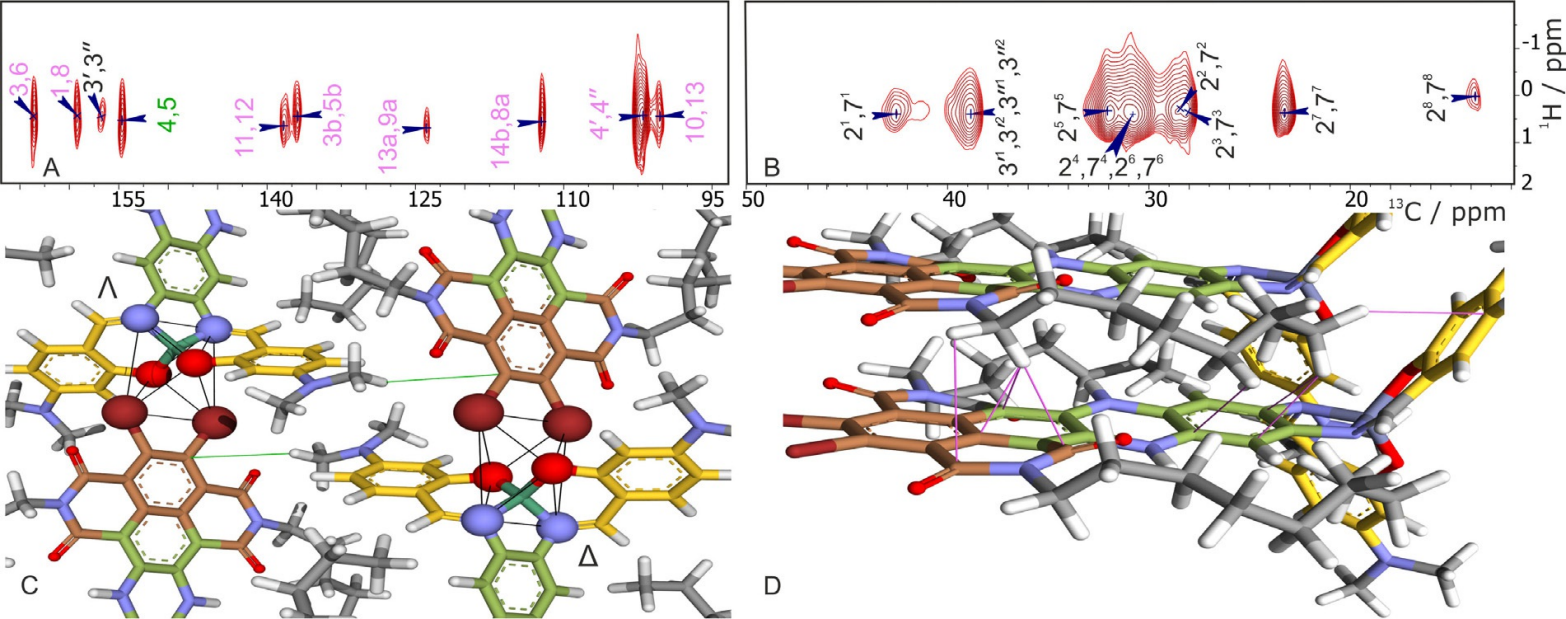
(A,B) Contour plot sections of ^1^H–^13^C correlation spectra collected from DATZnS(3′‐NMe) with a contact time of 4 ms; (C) the intermolecular interaction (green) between 4/5 ^13^C and dimethyl amine and the molecular recognition motif; (D) the folding of the tails is obtained from the transfer (violet) between phenazine ^13^C nuclei and the aliphatic ^1^H of the tails.

**Figure 4:**
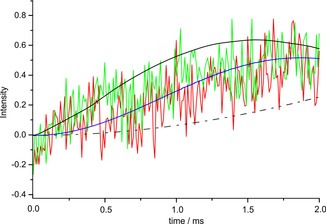
LGCP buildup curves of the polarization transfer to 4/5 (red) and 13*a*/9*a* (green) carbon nuclei compared with a simulated buildup for a heteronuclear ^1^H–^13^C spin pair separated by approximately 4 Å (blue). The buildup curves representing 3 Å (black solid lines) and 5 Å (black dotted lines) are also shown.

Strong π–π stacking interactions and aligned electric dipoles explained why the material had a high density, a low energy, and was insoluble. The aliphatic tails were oriented in the same direction as the salphen wings and were situated in voids between the phenazine moieties. Tight packing with the alkyl chains folded along the phenazine bridge of a neighboring molecule explained the observation of strong heteronuclear correlation signals between the abundant aliphatic ^1^H and rare phenazine ^13^C spins, providing efficient pathways for polarization transfer (Figure 3). The molecular recognition and distorted octahedral surrounding of the Zn^2+^ ion put the 3′‐NMe of salphen at 0.45 nm from the 4, 5 ^13^C nuclei in NDI, which was in quantitative agreement with the LGCP buildup kinetics and its simulation. This refinement represents the third step in our approach.

In our final and fourth step we determined the orientation of the material on the surface by simulating the diffraction pattern, thereby validating the indexing and space group. A view along the 0.69, 0, 0.69 lattice vector yielded the best match for the density and the diffraction pattern (Figure 2 C, D).^9^ The analysis validated the systematic absence of reflections from the *c*‐glide plane in the *P*2/*c* space group and showed that −1 0 1 and 1 0 −1 were quenched (Figure 2 B). The strong 0 1 0 and 0 −1 0 originated from lamellar spacing and alternating regions of Zn‐salphen and NDI. The phenazine dipoles were aligned along the surface and were perpendicular to the NDI stacks that ran parallel to the surface with the plane of the NDI rings at an angle of 45° (Figures 2 E, F). Figure 5 shows how molecular recognition leads to a transfer of molecular symmetry for scaffolding. Whereas steric hindrance favors screw axes or glide planes (symmetry operations with a translational component) to allow for interpenetration of symmetry‐related molecules, the screw axis is apparently suppressed in DATZnS(3′‐NMe) in favour of a twofold axis to accommodate intramolecular *C*_2_ symmetry (Figure 5 A). This is possible because of the rich structural variability introduced with the nonplanar metal salphen. It allows for packing in an achiral *P*2/*c* space group with a racemic mixture of the two enantiomeric species, thereby circumventing the need for a screw axis in favour of a *c*‐glide plane with inversion symmetry in the structure.

**Figure 5:**
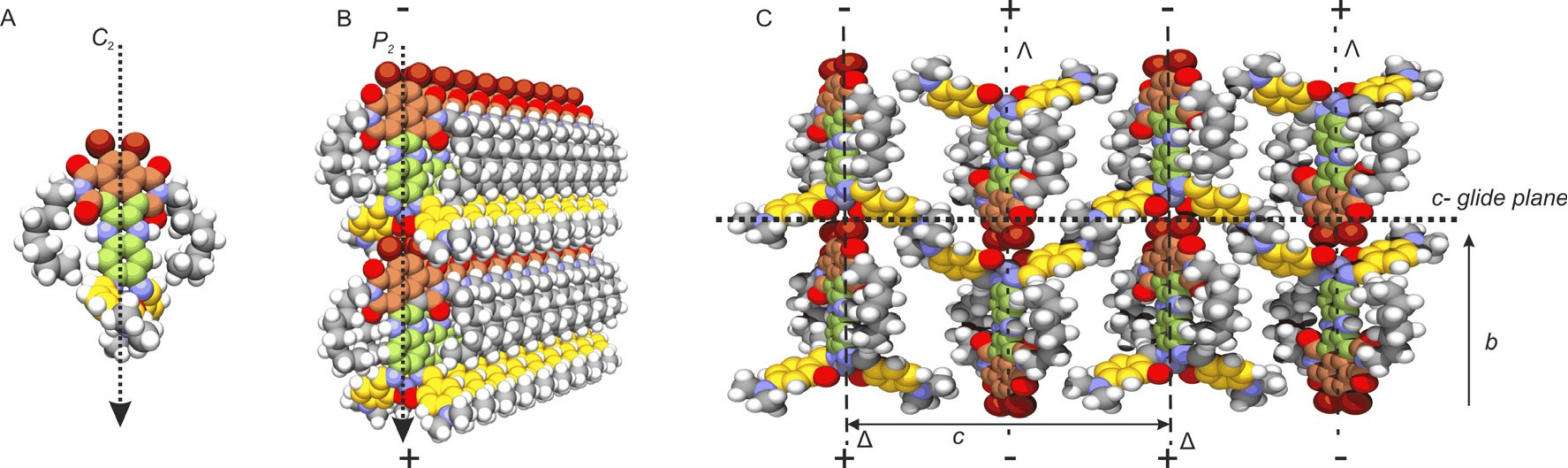
High‐resolution 3D lattice model for *para*‐crystalline DATZnS(3′‐NMe); (A) Molecular recognition for steering the packing starts from chiral building blocks with *C*_2_ symmetry. (B) These self‐assemble into enantiomerically pure Δ and Λ polar layers with a transfer of molecular *C*_2_ symmetry into supramolecular *P*2 symmetry. The layers comprise arrays of aligned dipoles with a positively charged salphen and negatively charged Br. (C) Alternating layers with opposite chirality self‐assemble with a *c*‐glide plane to release steric hindrance and establish a dense packing with quenching of electric dipoles.

With *C*_2_ molecular symmetry preserved, DATZnS(3′‐NMe) self‐assembles into polar planes without inversion symmetry elements, thereby mimicking the parallel stacking in the chlorosome antenna (Figure 5 B), for which the selectivity induced by chirality emerges at the salphen motif. The electric dipoles align and form extended arrays with a positive and a negative side to support charge separation following light absorption in the NDI columns running perpendicular to the electric‐field direction (Figure 5). DATZnS(3′‐NMe) forms extended chiral layers in the proposed 3D model, arising from planar arrangements of individual *C*_2_ motifs (known as organizational chirality), in which the net dipole moment is canceled owing to antiparallel layers.

In conclusion, we have demonstrated hybrid MAS NMR spectroscopy and TEM for de novo structure determination of a bioinspired *para*‐crystalline material. The concept can be further developed with, for example, pattern recognition across TEM and NMR datasets to facilitate the applicability and broaden the scope. This paves the way for structure determination of advanced organic supramolecular materials that bridge the gap from dead to living matter and are inaccessible to high‐resolution diffraction methods.

## Conflict of interest

The authors declare no conflict of interest.

## Supporting information

As a service to our authors and readers, this journal provides supporting information supplied by the authors. Such materials are peer reviewed and may be re‐organized for online delivery, but are not copy‐edited or typeset. Technical support issues arising from supporting information (other than missing files) should be addressed to the authors.

Supplementary
