# Supplementary material for: A Hybrid Solid‐State NMR and Electron Microscopy Structure‐Determination Protocol for Engineering Advanced para‐Crystalline Optical Materials
Source: Chemistry. 2017 Feb 16;23(14):3280–4. doi: 10.1002/chem.201700324 (PMC5347911; doi:10.1002/chem.201700324)
Supplement: Supplementary file 1 — Supplementary [file CHEM-23-3280-s001.pdf]

CHEMISTRY
A European Journal

Supporting Information

A Hybrid Solid-State NMR and Electron Microscopy Structure-Determination Protocol for Engineering Advanced *para*-Crystalline Optical Materials

Brijith Thomas,[a] Jeroen Rombouts,[b] Gert T. Oostergetel,[c] Karthick B. S. S. Gupta,[a] Francesco Buda,[a] Koop Lammertsma,[b, d] Romano Orru,[b] and Huub J. M. de Groot*[a]

chem_201700324_sm_miscellaneous_information.pdf

Supporting Information

Table of contents

1) 1D ^{13}C NMR 2
2) Structure of DATZnSTP homologue 2
3) ^{13}C 2D spectra at a short mixing time 3
4) LGCP build curve 3
5) TEM images 4
6) Input used for SIMPSON simulation 5
7) Experimental procedure 6
8) SSNMR spectra 7
9) TEM Measurement 7
10) Modelling and computational details 8
11) ^{13}C solid state chemical shiftsof the DATZnS(3′-NMe) 8
12) ^{13}C solution chemical shifts of the DATZnSTP 9
13) References 10

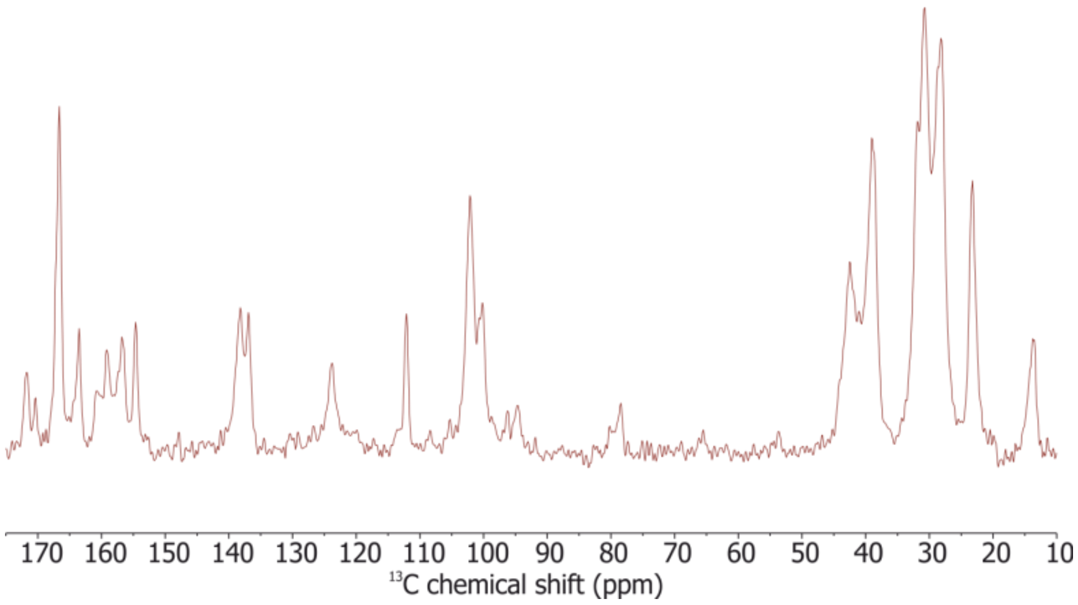

Figure S1. 1D ^{13}C CP-MAS spectrum of DATZnS(3′-NMe). The data were collected in 3092 scans with a MAS rotation frequency of 11 kHz. Spinning side bands were identified by collecting spectra at different spinning frequencies. 1D CPMAS NMR data collected from DATZnS(3′-NMe) reveal a good dispersion of ^{13}C signals.

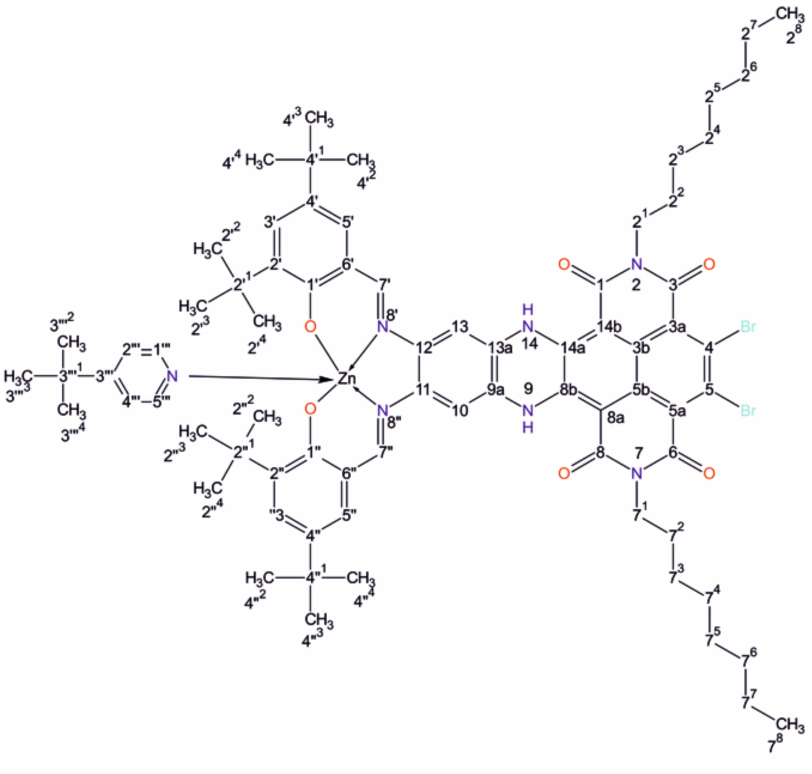

Figure S2. Structure of DATZnSTP homologue used for the solution state NMR assignment. Chemical shifts of corresponding carbon atoms are explained in Table S2.

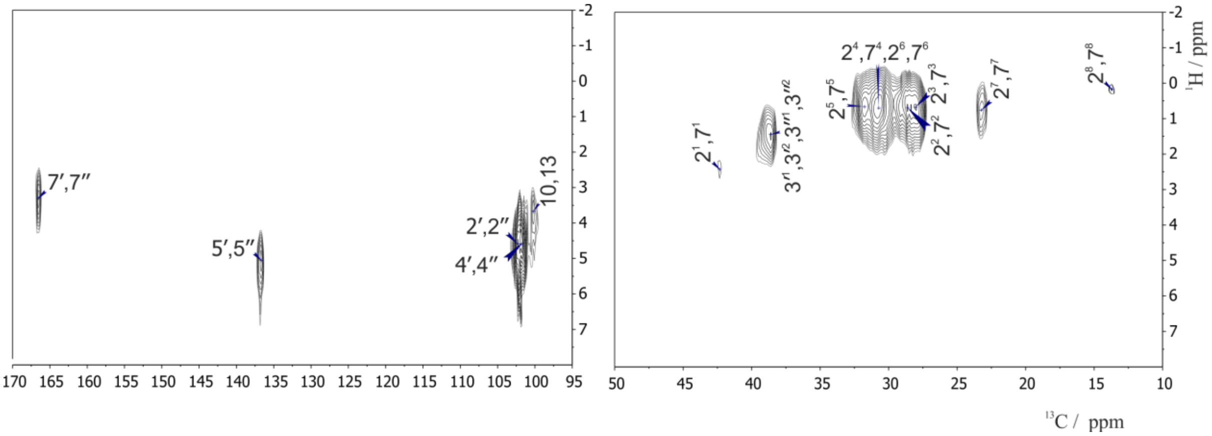

Figure S3 ^{13}C 2D spectra at a short mixing time.

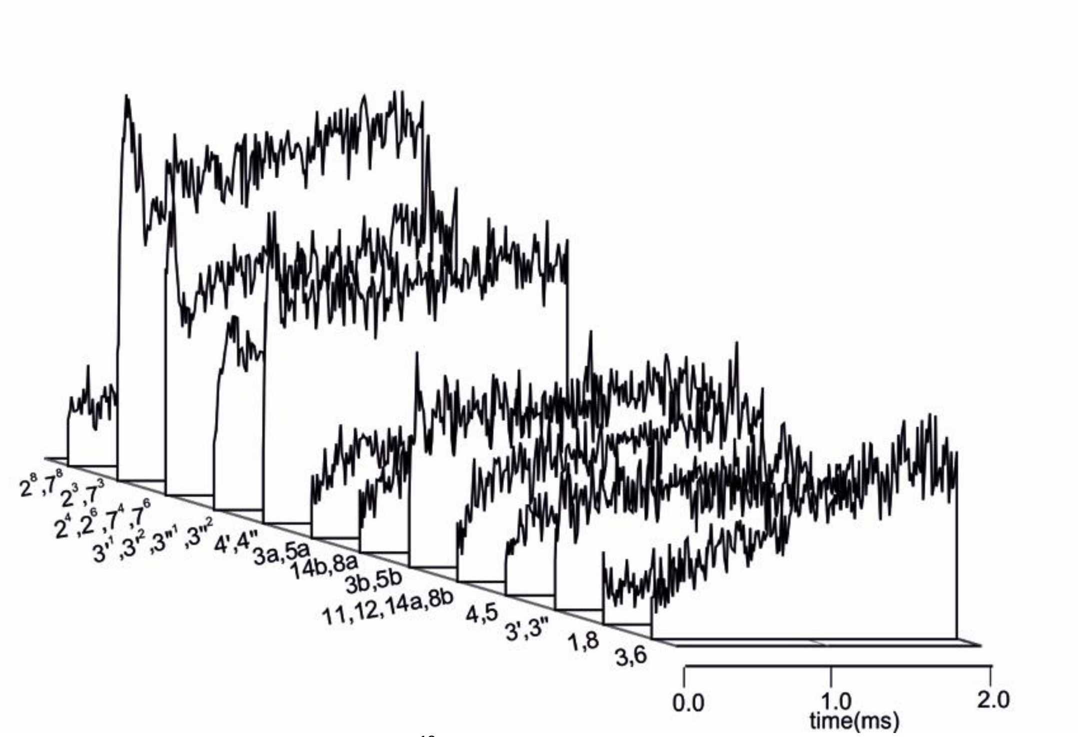

Figure S4. LGCP build curve plotted for selected ^{13}C nuclei.

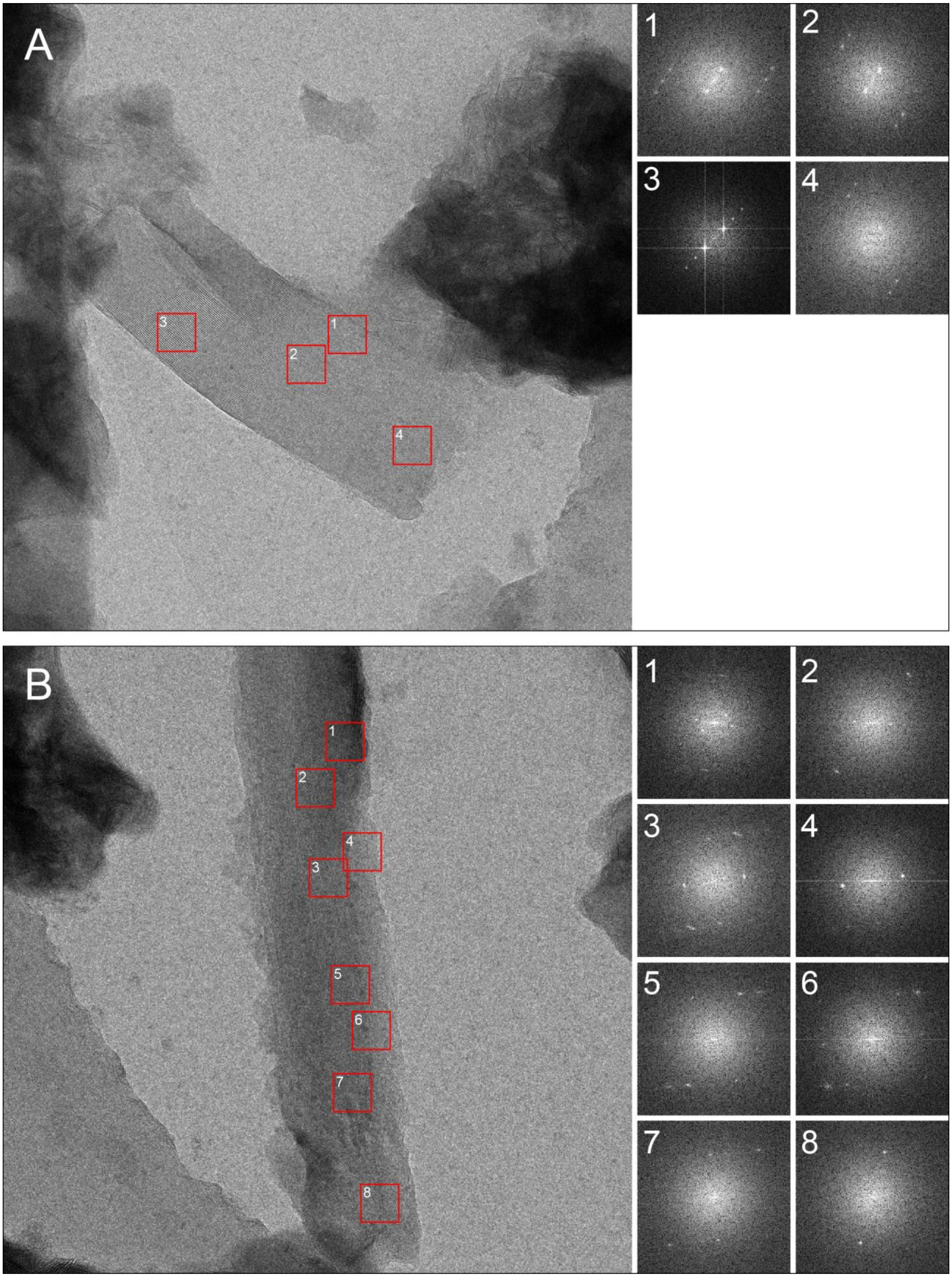

Figure S5. TEM images of DATZnS(3′-NMe) crystals in two different orientations (A and B) and their local Fourier transforms showing multiple reflection spots. Most of the reflections are higher order reflections of 0.547 nm, 1.685 nm, and 2.517 nm. The higher order reflections are in line with the *h*,0,*l*, *l*=2*n* reflection condition for a *c*-glide and a *P*2/*c* paracrystalline packing. Out of the multiple reflection spots, few are showing the periodic repetition of 0.74 nm (B 5), which indicates the possibility of polymorphism.

S6 SIMPSON simulation

LGCP build up curve simulations were performed for ^{1}H–^{13}C spin systems using the open-source simulation software SIMPSON with REPULSION powder averaging over' a set of 66 Euler angles with 8 γ angles at 11 kHz spinning and a static field corresponding to 750 MHz for ^{1}H.

Input used for SIMPSON simulation

```
spinsys {
  channels 1H 13C
        nuclei 1H  13C
        dipole 1 2 -472  0 0 0
}
par {
  method      direct
        start_operator  Inz-I2z
        detect_operator I2p
        crystal_file    rep66
        spin_rate       11000
        np              512
        gamma_angles    8
        sw              1000000
  variable    tsw 1.0e6/sw
}
proc pulseq {} {
  global par
  pulseid 5 50000 y 0 0
  acq_block {
    pulse $par(tsw) 50000 x 40000 x
        }
}
proc pulseq_lg {} {
        global par
        set effH 50000
  set taup [expr 54.74/90.0*5]
  pulseid $taup 50000 y 0 0
  set offset [expr $effH/sqrt(3.0)]
```

```
            set rfH [expr $effH/sqrt(1.5)]

  offset $offset 0

  acq_block {

    pulse $par(tsw) $rfH x 40000 x

            }

}

proc main {} {

  global par

            set f [fsimpson]

            fsave $f $par(name).fid -binary

  set par(pulse_sequence) pulseq_lg

  set f [fsimpson]

            fsave $f $par(name)_lg.fid -xreim

}
```

S7 Experimental procedure

For the purpose of our study we use Zinc(II) 6,6'-((1E,1'E)-((4,5-dibromo-2,7-dioctyl-1,3,6,8-tetraoxo-1,2,3,6,7,8,9,14-octahydro-[3,8] phenanthrolino [1,10-abc] phenazine-11,12-diyl) bis (azanylylidene)) bis (methanylylidene))bis(3-(dimethylamino)phenolate) (DATZnS(3'-NMe)) molecules (Figure 1). The DATZnS scaffold is a hybrid material with a core expanded naphthalene di- imide (cNDI) supramolecularly coupled with a Zinc bis- salicyimide phenylene (salphen) moiety and can be functionalized with 3'-NMe or 2',4' - tBu functional groups (Figure S7.1).[1] For the synthesis, anhydrous N,N- dimethylformamide (DMF) was obtained from Sigma-Aldrich and used as received. Melting points were measured using ΔT = 1 °C min^{-1} on a Stuart Scientific SMP3 melting point apparatus and are uncorrected. Infrared (IR) spectra were recorded using a Shimadzu FTIR-8400s spectrophotometer and wavelengths are reported in cm^{-1}. Unless stated otherwise, all reagents were used as received from commercial vendors.

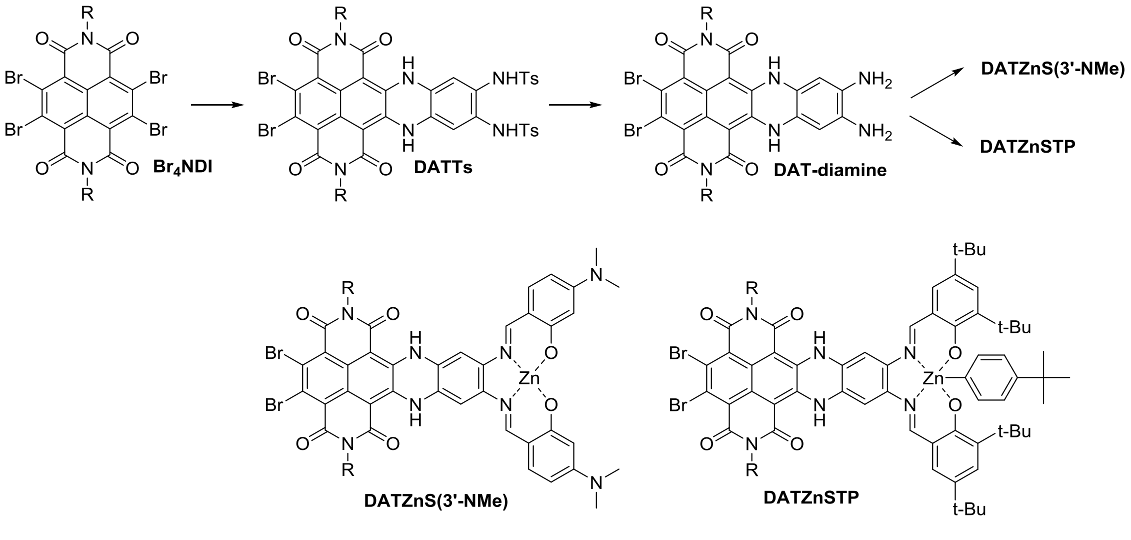

Figure S7.1 Overview of synthetic strategy towards DATZnS-dyads. R = *n*-octyl.

Synthesis of DAT-diamine: In a small round-bottomed flask, 15 mL of 98% H_2SO_4 was degassed for 15 min using an N_2 stream. DATTs[2] was added as a solid (131 mg, 0.12 mmol) and the resulting turquoise solution was stirred under N_2 atmosphere for 4 days. The reaction mixture was transferred under vigorous stirring into a 1 L flask containing 500 mL of H_2O and 200 mL of dichloromethane, after which $NaHCO_3$ was added as a saturated aqueous solution until gas evolution ceased. The organic phase was separated and concentrated under vacuum. A dark blue solid (DAT-diamine) was obtained in quantitative yield (85 mg). This

solid readily aggregates in solution, prohibiting recording of ^{1}H and ^{13}C solution NMR spectra. FTIR: 3456, 3416, 3362, 3339, 2953, 2914, 2849, 1678, 1570, 1489, 1452, 1425, 1286, 1234, 1134, 1094, 1013, 874, 812, 660 cm^{-1}. The first four frequencies are indicative of the presence of aniline-like N-H stretching vibrations.

Synthesis of DATZnS(3'-NMe): DAT-diamine intermediate (142 mg, 0.18 mmol) was prepared from DATTs, was dissolved in 15 mL dry, degassed DMF under an Ar atmosphere and heated to 110 °C in the dark. In a separate flask, 4-dimethylaminosalicylaldehyde (60 mg, 0.37 mmol) and zinc acetate dihydrate (320 mg, 1.75 mmol) were dissolved in dry, degassed DMF (5 mL) and kept under Ar. This mixture was stirred for 5 minutes, and added to the hot DMF solution via a syringe. After 4 hours, the reaction mixture was cooled down to room temperature and diluted with 25 mL saturated aqueous $NaHCO_3$ solution to induce precipitation. The dark blue precipitate was collected on a filter and washed with water, ethanol and chloroform to afford (after vacuum drying) 221 mg of a dark blue solid (95 % yield from DAT-diamine). IR (ATR FTIR): 3497, 3371, 2923, 2856, 1684, 1610, 1570, 1560, 1448, 1431, 1375, 1358, 1279, 1246, 1182, 1151, 843, 669, 660, 661, 584 cm^{-1}. Mp: > 300° C

S8 MAS NMR spectra

NMR spectra with high sensitivity and high resolution were obtained by exploiting heteronuclear cross-polarization (CP) along with magic angle spinning (CPMAS), in which polarization is transferred from abundant ^{1}H spins, to the dilute ^{13}C nuclei, followed by the observation of the signals from the carbon. MAS NMR experiments were performed with a Bruker AV-750 spectrometer equipped with a 4 mm triple resonance MAS probe head, using a ^{13}C radio frequency of 188.6 MHz and data were collected at a sample temperature of 298 K. The ^{79}Br resonance from KBr was used to set the magic angle. Chemical shifts are relative to TMS. Since the DATZnS(3′-NMe) and its DAT-diamine precursor (Figure S2) are insoluble, the NMR spectrum was assigned by homology to the response from a related compound, DATZnSTP (Table S2). A spinning frequency of 11 kHz ± 5Hz was used for the 2D ^{1}H-^{13}C heteronuclear correlation experiments. The ^{1}H spins were decoupled during acquisition using the two-pulse phase modulation TPPM scheme. Two-dimensional ^{1}H-^{13}C heteronuclear correlation data sets were obtained with the Phase-Modulated Lee-Goldburg (PMLG) experiment with a short CP time of 0.256 ms and a long CP time of 4 ms (Figure S8.1.A). The ^{1}H chemical shift was calibrated from a HETCOR dataset collected from tyrosine.HCl salt. A FSLG scale factor of 0.571 reproduces the ^{1}H chemical shifts for Tyr in solution. To collect the data set for the LGCP build up curves the sequence in Figure S2b was used. LG conditions were applied during CP and the contact time was increased from 0.1 to 2 ms.[3] NMR data were processed using the TopSpin 3.2 software (Bruker, Billerica, MA). OriginPro 9.1 (OriginLab Corporation, Northampton, MA) was used to process the LGCP build up curves for the selected ^{13}C nuclei and to do the Fourier transformation

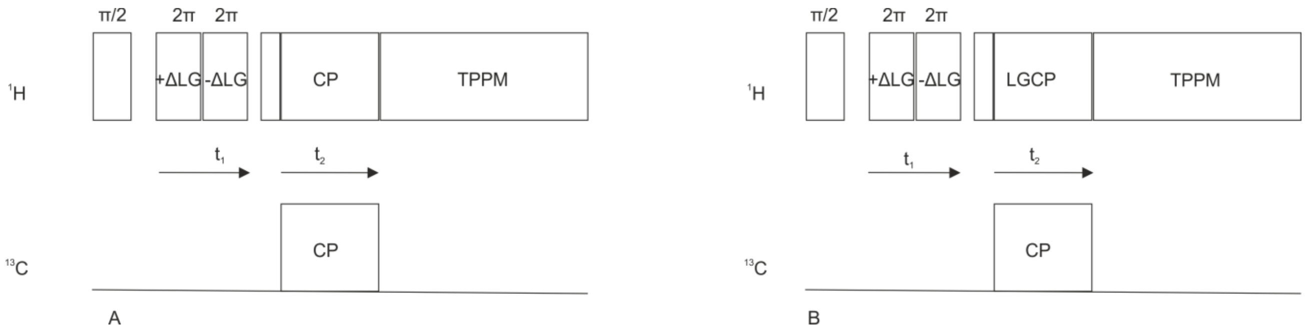

Figure S8.1. Pulse sequence used for (A) ^{1}H-^{13}C Hetcor experiment (B) for LGCP build up curve experiment.

S9 TEM Measurement

Samples for CryoEM were prepared by gently “crushing” the material with a small spatula onto a glass slide. It was suspended in ethanol and after settling a droplet from the upper half of the suspension containing the smaller fragments was put onto a carbon-coated grid, blotted, and dried in air. The samples were cooled down to -180° in the microscope and imaged with 300 kV electrons. Electron microscopy was performed with a Titan Krios electron microscope (FEI, Hillsboro, OR) at the NeCEN facility (Leiden, The Netherlands), at 47,000· nominal magnification(pixel size 0.147 nm at the level of the specimen). Images were recorded with a Falcon direct electron detector (FEI) at approx. 800 nm defocus and a dose of 2500 e^{-}/nm^{2}. 3D electron density was simulated from atomic coordinates using the EMAN pdb2mrc program.[4] The electron density map was projected onto a plane using the EMAN proc3d program to get a simulated EM image. Simulation of the TEM diffraction was performed with SingleCrystal (Crystalmaker Software Ltd, Oxford) after orienting the crystal structure in the CrystalMaker (Crystalmaker Software Ltd, Oxford) software.[5] Electron density map was generated with the EMAN electron microscopy processing suite, transferred into an electron density map and then projected onto a plane. For the simulation of the diffraction pattern the model was read into CrystalMaker, oriented, and projected onto the TEM diffraction pattern in SingleCrystal.[5a]

S10 Modelling and computational details

Computational modeling was performed with the Biovia Materials Studio Suite (Biovia, San Diego, CA).[6] Racemic packings were obtained for different space groups. A set of unit cells with a twofold axis was selected to accommodate the C_2 symmetry. A monomer was optimized with the Dreiding force field in FORCITE, and refined with $DMol^3$ to determine the atomic charges. The total charge was set to zero and the molecule was then positioned with the C_2 molecular axis aligned onto the twofold symmetry axis in the unit cell, second setting with *B*-Unique, Cell1 in Materials Studio. The monomer was shifted and rotated to minimize steric hindrance. The four symmetry operations then generate one pair of enantiomers, and only one set of enantiomers was kept. With the density at 1.67 g/cm^3, the position along the twofold axis was varied to minimize steric hindrance. The structure was then optimized using the FORCITE module with the Dreiding force field and ESP charges calculated with $DMol^3$ for the molecule in its C_2 configuration. Calculations were performed with $DMol^3$ as implemented in the Materials Studio package. The generalized gradient approximation (GGA) with the Perdew–Burke–Ernzerhof (PBE) functional was employed. In this work, the double numerical atomic orbital augmented by a polarization p-function (DNP) is chosen as the basis file 3.5. Global orbital cutoff value is 3.9 Å.

NMR chemical shift calculations for monomers were performed by optimizing the structure using the Gaussian 03 software package (Gaussian, Inc., Wallingford, CT) with the Becke, Lee, Yang, and Parr (BLYP) exchange-correlation functional with 6-311G basis set and using the NMR module in Gaussian.[7] The *P*2/*c* has the highest density and lowest lattice energy among the packings with other possible space groups. We performed a polymorph analysis with Materials studio to verify the possibility of other packings with higher density and lower energy across the five most frequent space groups, which we were not able to find.[8]

Table S1: ^{13}C solid state chemical shifts ($\sigma^{C}_{DATZnS(3'\text{-}NMe),\ expt}$) and calculated chemical shifts ($\sigma^{C}_{DATZnS(3'\text{-}NMe),\ calc}$) of the DATZnS(3′-NMe)

Position	$\sigma^{C*}_{DATZnS(3'\text{-}NMe),\ expt}$	$\sigma^{C}_{DATZnS(3'\text{-}NMe),\ calc}$
4, 5	154.6, 154.6	152.5, 152.5
3a, 5a	23.8, 123.8	125.6, 125.3
3, 6	166.6, 166.6	162.9, 162.9
3b, 5b	136.9, 136.9	130.0, 130.0
14b, 8a	112.1, 112.1	101.3, 101.3
13a, 9a	123.8, 123.8	129.0, 129.0
13, 10	100.1, 100.1	99.8, 99.8
12, 11	138.1, 138.1	142.1, 142.1
7', 7''	166.6, 166.6	150.2, 150.2
6', 6''	123.8, 123.8	122.7, 122.7
5', 5''	136.9, 136.9	138.2, 138.2
3', 3''	156.7, 156.7	154.1, 154.1
4', 4''	102.1, 102.1	109.1, 109.1
2', 2''	102.1, 102.1	108.5, 108.5
1', 1''	171.7, 171.7	176.7, 176.7
2^1 ,7^1	42.4, 42.4	46.9, 46.9
2^2, 7^2	28.6, 28.6	32.3, 32.3
2^3, 7^3	28.1, 28.1	34.5, 34.5
2^4, 7^4	30.7, 30.7	36.7, 36.7
2^5, 7^5	31.8, 31.8	35.1, 35.1
2^6, 7^6	30.7, 30.7	41.8, 41.8
2^7, 7^7	23.2, 23.2	29.3, 29.4
2^8, 7^8	13.7, 13.7	17.8, 17.8
$3'^{1}$ $3'^{2}$, $3''^{1}$, $3''^{2}$	39.0, 39.0, 39.0, 39.0	43.3, 43.3, 43.4, 43.4

Table S2: ^{13}C solution ($\sigma^{C}_{DATZnSTP,\ expt}$) and calculated chemical shifts ($\sigma^{C}_{DATZnSTP,\ calc}$) of the DATZnSTP.

Position	$\sigma^{C}_{DATZnSTP,\ expt}$	$\sigma^{C}_{DATZnSTP,\ calc}$
4, 5	140.6, 140.6	152.5, 152.5
3a, 5a	121.7,121.7	125.8,126.3
3,6	160.0,160.0	163.4,163.5
3b,5b	127.3,127.3	130.5,129.9
14b, 8a	95.5, 95.5	101.9, 102.5
1, 8	164.6, 164.6	167.0, 166.8
14a, 8b	127.5, 127.5	139.0, 138.7
13a, 9a	125.7, 125.7	129.8, 129.8
13, 10	102.9, 102.9	104.4, 102.1
12, 11	139.5, 139.5	146.8, 145.7
7', 7''	162.0, 162.0	158.4, 156.4
6', 6''	118.9, 118.9	127.7, 127.1
5', 5''	129.7, 129.7	136.5, 132.0
4', 4''	135.3, 135.3	142.6, 143.0
3', 3''	130.7, 130.7	133.1, 135.8
2', 2''	143.3, 143.3	148.8, 151.5
1', 1''	173.4, 173.4	177.5, 177.4
$4'^{1}$, $4''^{1}$	34.2, 34.2	44.4, 45.2
$4'^{2}$, $4''^{2}$	31.8, 31.8	35.3, 33.6
$4'^{3}$, $4''^{3}$	31.8, 31.8	35.1, 30.4
$4'^{4}$, $4''^{4}$	31.8, 31.8	30.8, 33.7
$2'^{1}$, $2''^{1}$	36.3, 36.3	47.7, 46.9
$2'^{2}$, $2''^{2}$	30.2, 30.2	34.6, 34.8
$2'^{3}$, $2''^{3}$	30.2, 30.2	30.5, 29.3
$2'^{4}$, $2''^{4}$	30.2, 30.2	30.1, 29.5
1''', 5'''	149.2, 149.2	149.6, 153.9
5'''	149.2	153.91
2''', 4'''	121.4, 121.4	127.8, 126.0
3'''	161.3	168.2
$3'''^{1}$	34.4	45.4
$3'''^{2}$, $3'''^{3}$, $3'''^{4}$	30.0, 30.0, 30.0	34.1, 34.4, 29.4
2^{1}, 7^{1}	41.5, 41.5	48.4, 49.1
2^{2}, 7^{2}	28.3, 28.3	33.3, 33.1
2^{3}, 7^{3}	27.8, 27.8	32.1, 31.9
2^{4}, 7^{4}	29.8, 29.8	36.6, 41.1
2^{5}, 7^{5}	29.8, 29.8	29.3, 31.6
2^{6}, 7^{6}	32.2, 32.2	37.4, 43.1
2^{7}, 7^{7}	23.1, 23.1	25.0, 32.6
2^{8}, 7^{8}	14.4, 14.4	14.6, 18.6

References

[1] J. A. Rombouts, J. Ravensbergen, R. N. Frese, J. T. M. Kennis, A. W. Ehlers, J. C. Slootweg, E. Ruijter, K. Lammertsma, R. V. A. Orru, *Chemistry – A European Journal* **2014**, *20*, 10285-10291.

[2] N. Banerji, S. V. Bhosale, I. Petkova, S. J. Langford, E. Vauthey, *Physical Chemistry Chemical Physics* **2011**, *13*, 1019-1029.

[3] B. J. van Rossum, C. P. de Groot, V. Ladizhansky, S. Vega, H. J. M. de Groot, *Journal of the American Chemical Society* **2000**, *122*, 3465-3472.

[4] S. J. Ludtke, P. R. Baldwin, W. Chiu, *Journal of Structural Biology* **1999**, *128*, 82-97.

[5] aY. W. Yin, *Journal of the American Chemical Society* **2004**, *126*, 14996-14996; bS. C. Kohn, *Terra Nova* **1995**, *7*, 554-556.

[6] J. D. Evans, D. M. Huang, M. Haranczyk, A. W. Thornton, C. J. Sumby, C. J. Doonan, *CrystEngComm* **2016**, *18*, 4133-4141.

[7] M. J. Frisch, G. W. Trucks, H. B. Schlegel, G. E. Scuseria, M. A. Robb, J. R. Cheeseman, G. Scalmani, V. Barone, B. Mennucci, G. A. Petersson, H. Nakatsuji, M. Caricato, X. Li, H. P. Hratchian, A. F. Izmaylov, J. Bloino, G. Zheng, J. L. Sonnenberg, M. Hada, M. Ehara, K. Toyota, R. Fukuda, J. Hasegawa, M. Ishida, T. Nakajima, Y. Honda, O. Kitao, H. Nakai, T. Vreven, J. A. Montgomery Jr., J. E. Peralta, F. Ogliaro, M. J. Bearpark, J. Heyd, E. N. Brothers, K. N. Kudin, V. N. Staroverov, R. Kobayashi, J. Normand, K. Raghavachari, A. P. Rendell, J. C. Burant, S. S. Iyengar, J. Tomasi, M. Cossi, N. Rega, N. J. Millam, M. Klene, J. E. Knox, J. B. Cross, V. Bakken, C. Adamo, J. Jaramillo, R. Gomperts, R. E. Stratmann, O. Yazyev, A. J. Austin, R. Cammi, C. Pomelli, J. W. Ochterski, R. L. Martin, K. Morokuma, V. G. Zakrzewski, G. A. Voth, P. Salvador, J. J. Dannenberg, S. Dapprich, A. D. Daniels, Ö. Farkas, J. B. Foresman, J. V. Ortiz, J. Cioslowski, D. J. Fox, Gaussian, Inc., Wallingford, CT, USA, **2009**.

[8] Q. Zeng, Y. Qu, J. Li, H. Huang, *RSC Advances* **2016**, *6*, 5419-5427.
